# Unnecessary Workup of Asymptomatic Neonates in the Era of Group B Streptococcus Prophylaxis

**DOI:** 10.1155/2010/369654

**Published:** 2010-08-22

**Authors:** Brad Buckler, Jason Bell, Ralph Sams, William Cagle, Sue Anne Bell, Carla Allen, Don Sutherland, Jatinder Bhatia

**Affiliations:** ^1^Department of Pediatrics, Medical College of Georgia, Augusta, GA 30912, USA; ^2^Department of Obstetrics and Gynecology, Medical College of Georgia, Augusta, GA 30912, USA; ^3^Department of Sugery, Mercer University School of Medicine, Macon, GA 31207, USA; ^4^Georgia Prevention Instuite, Medical College of Georgia, Augusta, GA 30912, USA

## Abstract

Asymptomatic term neonates born to mothers who are Group B Streptococcus (GBS) unknown or GBS positive but “inadequately” treated prior to delivery do not require invasive laboratory evaluation. We conducted a retrospective cohort study of mother/baby dyads born from January 1, 2005 until September 30, 2007 at the Medical College of Georgia. Their current protocol is to obtain a Complete Blood Count with Differential (CBC with D), Blood Culture (BC), and C-reactive protein (CRP) after birth. Mother/baby dyads (*n* = 242) that met inclusion criteria were reviewed. Of these 242 babies 25 (10%) were started on antibiotics after the initial lab values were known. None of the blood cultures were positive and the CRP's were normal. The 2002 GBS guidelines call for laboratory evaluation of “at-risk” neonates, but the workup of these babies is not only costly, it does not provide any advantage over old fashioned clinical observation for the evaluation and treatment of early onset GBS sepsis.

## 1. Introduction

Group B Streptococcus (GBS) is a gram-positive cocci that may be found in the gastrointestinal tract, respiratory tract, urinary tract, and genital tract [1]. It is one of the most common causes of early onset neonatal sepsis, which occurs within the first 6 days of life. Approximately 30% of women have asymptomatic GBS colonization at some point during their pregnancy, and about 20% remain colonized at the time of birth [1]. GBS has nine serotypes that have been identified, which are differentiated by the polysaccharide capsule of the organism. Types I, II, and III are most commonly associated with neonatal sepsis, with type III being highly associated with central nervous system involvement [2]. 

 Asymptomatic term neonates born to mothers who are GBS unknown or GBS positive but “inadequately” treated prior to delivery do not require invasive laboratory evaluation [1, 3]. This evaluation may include a Complete Blood Count with Differential (CBC with D), a Blood Culture (BC), and a C-reactive protein (CRP). The Center for Disease Control (CDC) 2002 GBS guidelines call for a laboratory evaluation of “at risk” neonates, but they do not provide data to the usefulness of this practice [4]. 

 Laboratory evaluation of these babies is low yield, costly, and disrupts mother baby bonding soon after birth. These babies can be appropriately managed with careful clinical observation for signs and symptoms of sepsis. Our primary outcome was to determine the number of cases of sepsis that were diagnosed based on these screening practices.

## 2. Materials and Methods

We conducted a retrospective cohort study of mother/baby dyads born and cared for in the newborn nursery at the Medical College of Georgia (MCG) in Augusta, Georgia. Our study was from January 1, 2005 until September 30, 2007. During this time period 5,342 babies were born. Inclusion criteria consisted of babies born at term (>37 weeks completed gestation), mother's GBS status unknown or positive at the time of delivery, no antibiotics or antibiotics less than 4 hours prior to delivery, and a laboratory evaluation upon admission to the newborn nursery. There were no exclusion criteria. 

 The current protocol in the newborn nursery is to obtain a Complete Blood Count with a Differential (CBC with D), a Blood Culture (BC), and a C-reactive protein (CRP) immediately after birth. Then based on the results of these lab tests and the discretion of the attending the decision about whether to start antibiotics is made. 

 The study was approved by the Institution Review Board at the hospital.

## 3. Results

During the study period there were 242 mother/baby dyads that met the inclusion criteria. Of these 242 babies only 25 (10%) were started on antibiotics. The decision to start antibiotics was made by an attending general pediatrician based on the initial lab values. The antibiotic regimen was Ampicillin 100 mg/kg/dose every 12 hours and Gentamicin 4 mg/kg/dose every 24 hours. Antibiotic therapy was discontinued after 48 hours on 23 out of the 25 babies that were started on antibiotics, and the other 2 babies received 7 days of antibiotics for signs of clinical sepsis. Both of these babies were in the GBS positive No antibiotics group, and both were discharged in good health after completing their antibiotic course. None of the 242 babies had a positive blood culture and the C-reactive protein levels were normal.

## 4. Discussion

In this era of intrapartum prophylaxis for early onset GBS disease, we have seen a decline in the rate of early onset GBS disease from 0.6 per 1000 in live births in 2000 to 0.39 per 1000 live births in 2006 [5, 6]. The 2002 CDC guidelines have been effective at reducing the incidence of early onset GBS sepsis. However, the guidelines do not address or give support to the “limited evaluation” of asymptomatic babies. Safier et al. estimated that based on the current rate of GBS disease it would take about 10,000 blood cultures to identify 1 case of GBS sepsis [7]. Ottolini et al. found similar findings, as they did not have a positive blood culture in 1665 “at risk” term newborns [3]. In our institution, if a baby is delivered and the mother's GBS status is unknown or a maternal transfer without her complete medical record, then the obstetricians risk stratify the mother per the 2002 CDC guidelines [4]. If she does not meet the criteria for intrapartum prophylaxis, then she does not receive antibiotics. The problem arises in that the pediatricians treat an unknown GBS status as potential positive, and even though the mother appropriately does not receive antibiotics, the baby is subjected to a laboratory evaluation. The disconnect between the obstetricians and the pediatrician causes serious questions for these mothers who have just given birth. Many times the pediatricians tell the mother that since the GBS status was not known and she did not receive antibiotics that her baby will require an invasive laboratory evaluation. The mother then questions her obstetrician as to why she was not given antibiotics. This has led to obstetricians to give antibiotics to mothers who do not meet criteria based on the 2002 CDC guidelines in order to keep the pediatricians from subjecting asymptomatic neonates from this invasive evaluation. Another factor is the 4-hour window required for appropriate treatment of intrapartum antibiotics from the time the first does of antibiotics is given until the delivery [4]. More than half of our babies were evaluated solely based on the fact that the delivery occurred prior to this 4-hour cutoff. There have been several studies that have questioned this 4-hour window [8, 9]. Bloom et al. showed that bactericidal concentrations of ampicillin could be achieved within 5 minutes after infusion in both maternal and umbilical cord sera [10]. The New Zealand GBS Consensus Working Party that “well appearing babies born >35 weeks gestation to women with GBS risk factors who have received either no or inadequate (<4 hours) chemoprophylaxis should be observed closely in hospital for at least 24 hours” [11].

## 5. Conclusions

In conclusion, we feel that clinical observation, rather than an indepth laboratory workup is sufficient for the evaluation of asymptomatic, GBS “at-risk” neonates. The results of our study support the conclusion that subjecting asymptomatic neonates to multiple blood draws and invasive laboratory procedures is low yield. Furthermore, we feel it is costly and disrupts maternal/child bonding. We recommend, rather, that the clinician should implement serial examinations for the observation of sepsis. Laboratory exams should be initially withheld, though implemented if clinical suspicion warrants. We feel this approach is more cost effective and less invasive while providing the same level of care. Further studies are needed to provide data to further stratify the need for invasive examination of asymptomatic Group-B Strep “at-risk” neonates.

## Figures and Tables

**Table 1:** Number of babies treated based on mother's GBS status and doses of antibiotics given prior to delivery.

	No. of Babies	No. Babies treated with antibiotics	Positive cultures
GBS unknown no antibiotics	36	8	0
GBS unknown 1 dose antibiotics	25	1	0
GBS positive No antibiotics	51	5	0
GBS positive 1 dose Antibiotics	130	11	0

Totals	**242**	**25**	**0**

**Table 2:** Lab results for babies not treated with antibiotics after delivery and babies treated with antibiotics after delivery.

	No antibiotics	Yes antibiotics
	*n* = 217	*n* = 25
White blood cell count	19.2	20.9
I : T ratio	0.09	0.30
C-reactive protein	0.28	0.68

## References

[1] Tumbaga PF, Philip AG (2006). Perinatal Group B streptococcal infections and the new Guidelines: an update. *NeoReviews*.

[2] Anderson-Berry AL, Bellig LL (2008). Neonatal sepsis. *Emedicine*.

[3] Ottolini MC, Lundgren K, Mirkinson LJ, Cason S, Ottolini MG (2003). Utility of complete blood count and blood culture screening to diagnose neonatal sepsis in the asymptomatic at risk newborn. *Pediatric Infectious Disease Journal*.

[4] Schrag S, Gorwitz R, Fultz-Butts K, Schuchat A (2002). Prevention of perinatal group B streptococcal disease. Revised guidelines from CDC. *Morbidity and Mortality Weekly Report*.

[7] Saifer RA, Robins SH, Picone CM, Tafari N (2002). Blood culture screening of newborns at risk for early onset neonatal group B streptococcal disease. *Pediatric Research*.

[8] De Cueto M, Sanchez M-J, Sampedro A, Miranda J-A, Herruzo A-J, Rosa-Fraile M (1998). Timing of intrapartum ampicillin and prevention of vertical transmission of group B streptococcus. *Obstetrics and Gynecology*.

[9] McNanley AR, Glantz JC, Hardy DJ, Vicino D (2007). The effect of intrapartum penicillin on vaginal group B streptococcus colony counts. *American Journal of Obstetrics and Gynecology*.

[10] Bloom SL, Cox SM, Bawdon RE, Gilstrap LC (1996). Ampicillin for neonatal group B streptococcal prophylaxis: how rapidly can bactericidal concentrations be achieved?. *American Journal of Obstetrics and Gynecology*.

[11] Campbell N, Eddy A, Darlow B, Stone P, Grimwood K (2004). The prevention of early-onset neonatal group B streptococcus infection: technical report from the New Zealand GBS Consensus Working Party. *New Zealand Medical Journal*.

